# Beyond the Classroom: Inspiring Medical and Health Science Students to Learn Surface Anatomy

**DOI:** 10.1007/s40670-022-01521-0

**Published:** 2022-02-21

**Authors:** Claudia M. Diaz

**Affiliations:** School of Dentistry and Medical Sciences, Charles Sturt University, Albury, NSW 2640 Australia

**Keywords:** Extra-curricular, Body painting, Engagement, Learning, Innovation, Surface anatomy, Palpation, Physical examination

## Abstract

This qualitative and quantitative study offered students the opportunity to participate in engaging and inspiring activities “outside the classroom”, to extend their experience and knowledge of surface anatomy. Medical and health science students benefit from studying surface anatomy as it is relevant to their future professions that deal with patients and clients. Surface anatomy is an essential part of the learning process that allows students an opportunity to identify anatomical structures on living people and to develop their palpation and tactile skills for physical examinations of patients. Body painting is a student-centred, engaging, and motivating approach to learn surface anatomy in anatomy practical classes. In this study, anatomy learning was extended “beyond the classroom” through extra-curricular body painting projects. These projects were run by student teams consisting of a student model, student artists (4–5), and a student photographer, under the direction of the chief investigator. A total of sixteen body painting projects were carried out from 2010 to show the skeletal system, the muscular system, pregnancy, respiratory and gastrointestinal systems, and the neurovascular systems of the entire body. A SurveyMonkey of 31/41 active participants suggested that participants enjoyed the projects (94–100%), found them relevant to their future profession (80–87%), and considered them to assist with deeper understanding (94%) and long-term memory (93%) of anatomy. Learning anatomy outside the classroom through extra-curricular body painting projects was a successful way to engage, motivate, and inspire participants and first year anatomy students to study surface anatomy and to develop their physical examination skills.

## Introduction

Although there have been few reports of anatomy learning outside the classroom, a shift has been reported from didactic methods to self-directed and independent study in medical education. Musculoskeletal anatomy has been taught previously through yoga and pilates [1] and the use of collaborative and self-directed learning approaches to teach surface anatomy and peer physical examination were shown to have a positive effect [2]. Self-directed learning helps students to develop their self-reflection and life-long learning skills and active learning of anatomy allows students to interact with the learning process. In particular, it has been reported that active learning and life-long learning are the result of social interaction and discussions in a clinical context [3–5]. Additionally, it has been suggested that an increased need for more diverse learning has led to an evolution of teaching practices outside the classroom [6]. In the area of biology, it was shown that the implementation of special peer-led team learning workshops outside the classroom was successful in assisting students to understand the subject and to learn to communicate appropriate solutions [7]. Education outside the classroom has been reported in school children in Scandinavia and involved innovative teaching methods that focused on interdependent relations between factors such as social relations, well-being, and motivation [8].

Surface anatomy, which is the identification of anatomical structures on living human beings, has been studied using anatomical body painting (BP) at several universities as a novel approach to teach human anatomy [4, 9–18]. When studying human anatomy, it is important that students also focus their efforts on surface anatomy [19–21] that deals with observation of living bodies to assess form, proportions, and surface landmarks that relate to underlying, deeper structures [22]. The study of surface anatomy is important as it allows students the opportunity to consolidate their learning using cadavers and relate it to living bodies. Surface anatomy has the advantage that it can be done on anyone: oneself, classmates, colleagues, friends, or family. BP is engaging as it involves both drawing and the study of surface anatomy [22–24]. BP is an important tool for learning surface anatomy and clinical skills, and is important for future health professionals and clinicians that will be working with patients or clients and as such need to identify anatomical structures on real bodies. It is a highly captivating and fun approach for students to learn human anatomy by observing, identifying surface anatomy features, palpating, drawing, and then painting [4, 9–14, 16–18]. BP has also been used successfully in veterinary anatomy on horses [25].

Medical and health science students necessarily need to master the skills of robust communication, and effective palpation and tactile skills for the safety of their future patients [26]. Many students report discomfort with intimate physical examinations, so recommendations have been made for teaching techniques to initially include the use of inanimate simulation models. Students then develop competency and progression to clinical situations depending on the individual student’s skills and experience [26]. Learning of surface anatomy is crucial for identification of thoracic landmarks for appropriate patient care by thoracic surgeons [27]. A clinical anatomy practicum was used to prepare students for inspection, palpation, percussion, and auscultation of the cardiovascular, respiratory, abdominal, and urogenital systems and self-assessment reported an improvement in understanding of the anatomical basis for clinical examination [28]. In chiropractic teaching, the high number of hours dedicated to anatomy has been reported to assist clinical skills and the retention of knowledge [29]. More recently, ultrasound skills [30–33], video-assistance [34], e-learning [35], and mobile applications [36] have been used to assist and enhance students’ physical examination skills. However, it has been suggested that the development and reliance on advanced imaging has led to a deterioration of physical examination skills among medical students [33]. Although ultrasound training can assist student palpation skills [30, 31, 33], it was reported that students needed pre-course anatomy learning before the training [30]. Devi et al. [34] reported that traditional demonstration of palpation skills scored much better than the video-assisted teaching program suggesting that blended learning techniques may be the way forward for enhancing learning. “Hands-on” approaches for learning physical examination and palpation have been reported to improve the clinical skills and professional attitudes of students assisting their transition into clinical practice [37].

BP is a popular approach that is suitable for the development of physical examination skills in all health professional students and students of different cultures. This approach has been shown to be successful when implemented as part of the anatomy curriculum, and included in weekly anatomy practical classes. It has also been suggested that this approach may result in improved results for medical and health science students [3, 4]. Student performance is improved significantly by an active learning environment that encourages and teaches successful self-directed study strategies [4, 38]. This research differed from previous studies by investigating the value of teaching anatomy, through BP of the *entire* human body, “outside the classroom”. We attempted to inspire and drive learning by running *extra-curricular* BP projects to encourage self-directed, group-based learning, to facilitate the development of physical examination and palpation skills and to engage anatomy students further to learn surface anatomy outside the classroom. This work also aimed to produce learning resources and promotional materials for the department and the university.

## Methods

**Fig. 1:**
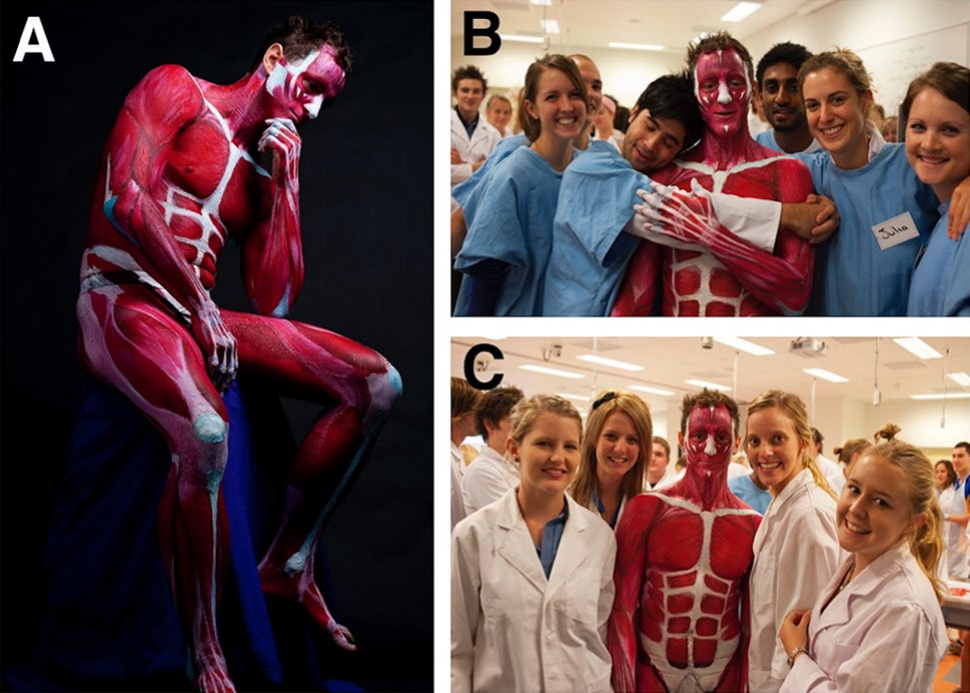
The first Anatomical Man at JCU project depicted muscles of the entire body and was carried out near the anatomy teaching laboratory. The completed Anatomical Man (**a**) was brought into the class to motivate and inspire both tutors (**b**) and students (**c**)

Anatomical BP projects by the chief investigator were carried out at James Cook University (JCU) (2008–2012) and Royal Melbourne Institute of Technology University (RMIT) (2012–2018). The BP projects were developed from 2010 onwards to assist with the introduction of BP during the first week of musculoskeletal anatomy as a motivational tool to encourage and inspire student engagement and learning “outside the classroom”. The projects were organised by the chief investigator and were carried out entirely by a student team consisting of a model, artists (4–5), and a photographer. The projects were not run during the regular teaching schedule, but rather were programmed as extra-curricular events, outside the classroom. In these projects, student artists painted the *entire* body of a student model to further their own anatomical knowledge and as a way of shocking, inspiring, and motivating their classmates. This was done within or near the teaching laboratory so that students could observe and participate if desired (Fig. 1). At least one to two projects were carried out each year. This research was approved by the RMIT Human Ethics Committee (ASEHAPP 16–13) for the student surveys. Students were asked to sign a media release form in week 1 and as part of the BP projects to allow the researcher to use all photographs.

### Participants

Participants in these projects were students who were doing, or had done, anatomy courses with the chief investigator. It was an important decision to use current anatomy students, rather than students from the art department as their knowledge of anatomy was crucial in producing accurate representations of the human body. All cohorts of students had talented student artists who were capable of this standard of work. Student participants volunteered or were recruited through word of mouth. Student models and photographers usually volunteered; student artists volunteered or were invited by the chief investigator after observing the quality of their work in anatomy classes. Students who participated in these projects were from the medical, pharmacy, engineering, and dentistry programs at JCU or from the chiropractic, osteopathy, and biomedical science programs at RMIT.

### Procedure

**Fig. 2:**
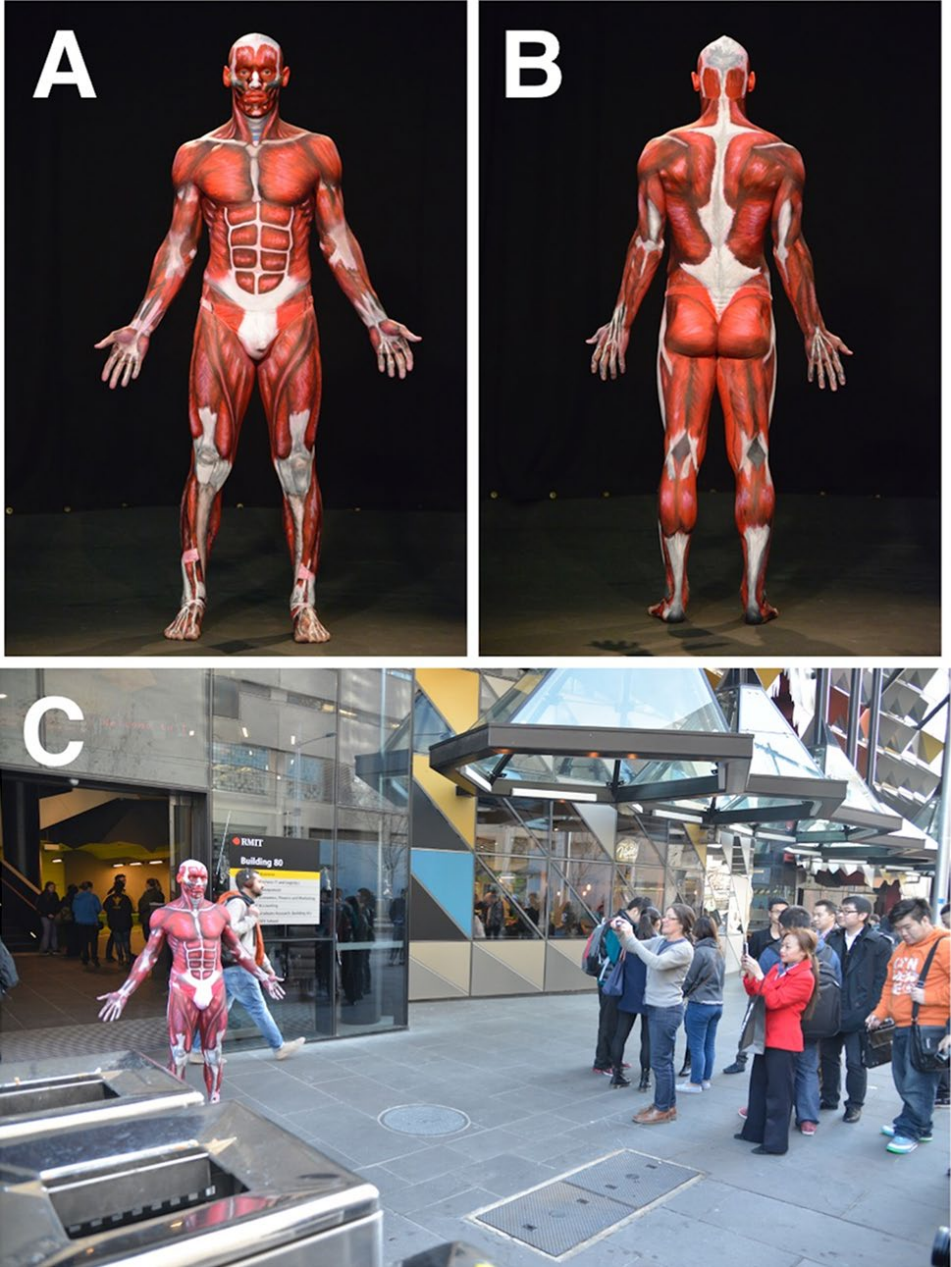
The finished Anatomical Man project in Melbourne, Australia; front (**a**) and back (**b**). The completed Anatomical Man was taken out onto a major Melbourne street adjacent to the university (**c**)

**Fig. 3:**
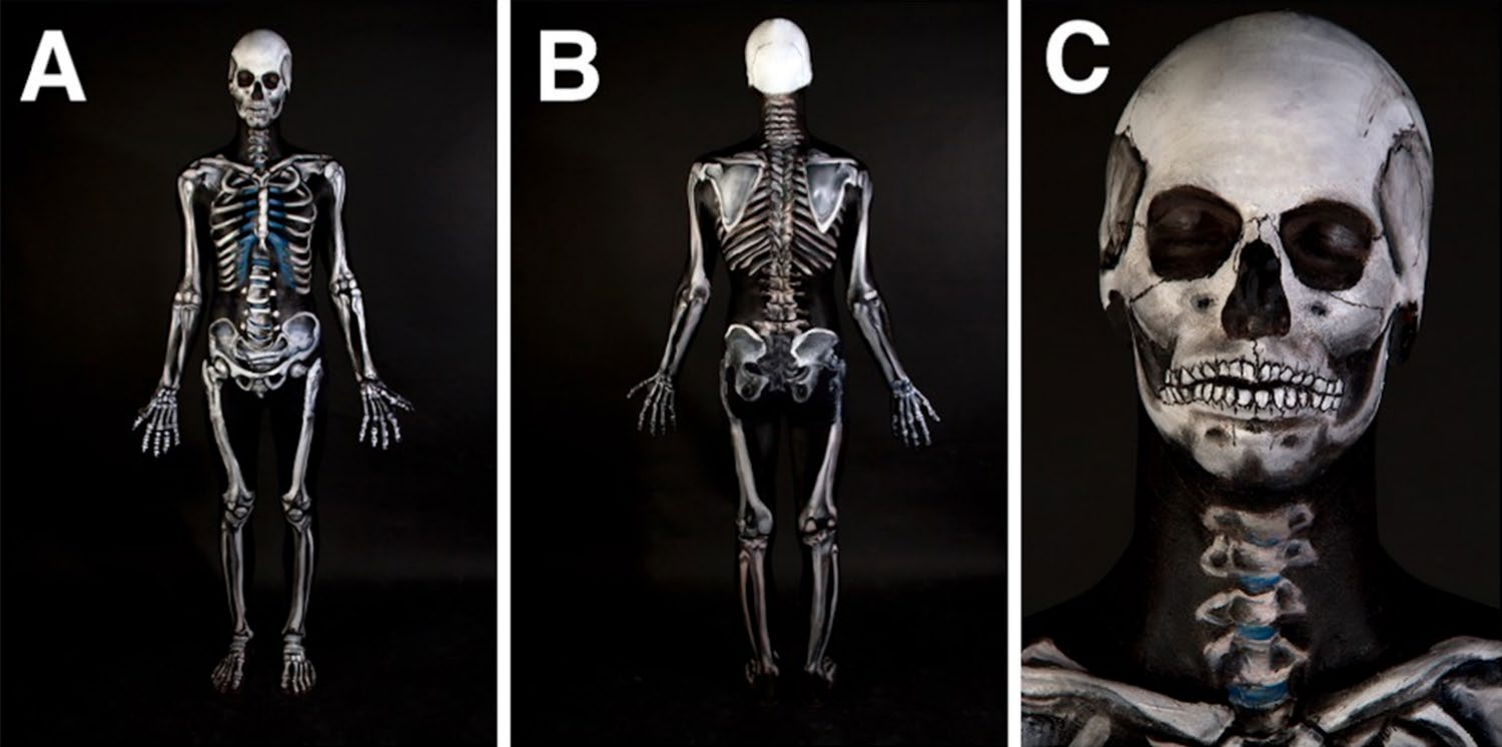
The completed Skeletal Man project at JCU displayed all the bones of the body on a background of black skin. Front (**a**), rear (**b**), and head view (**c**) of the body are illustrated

**Fig. 4:**
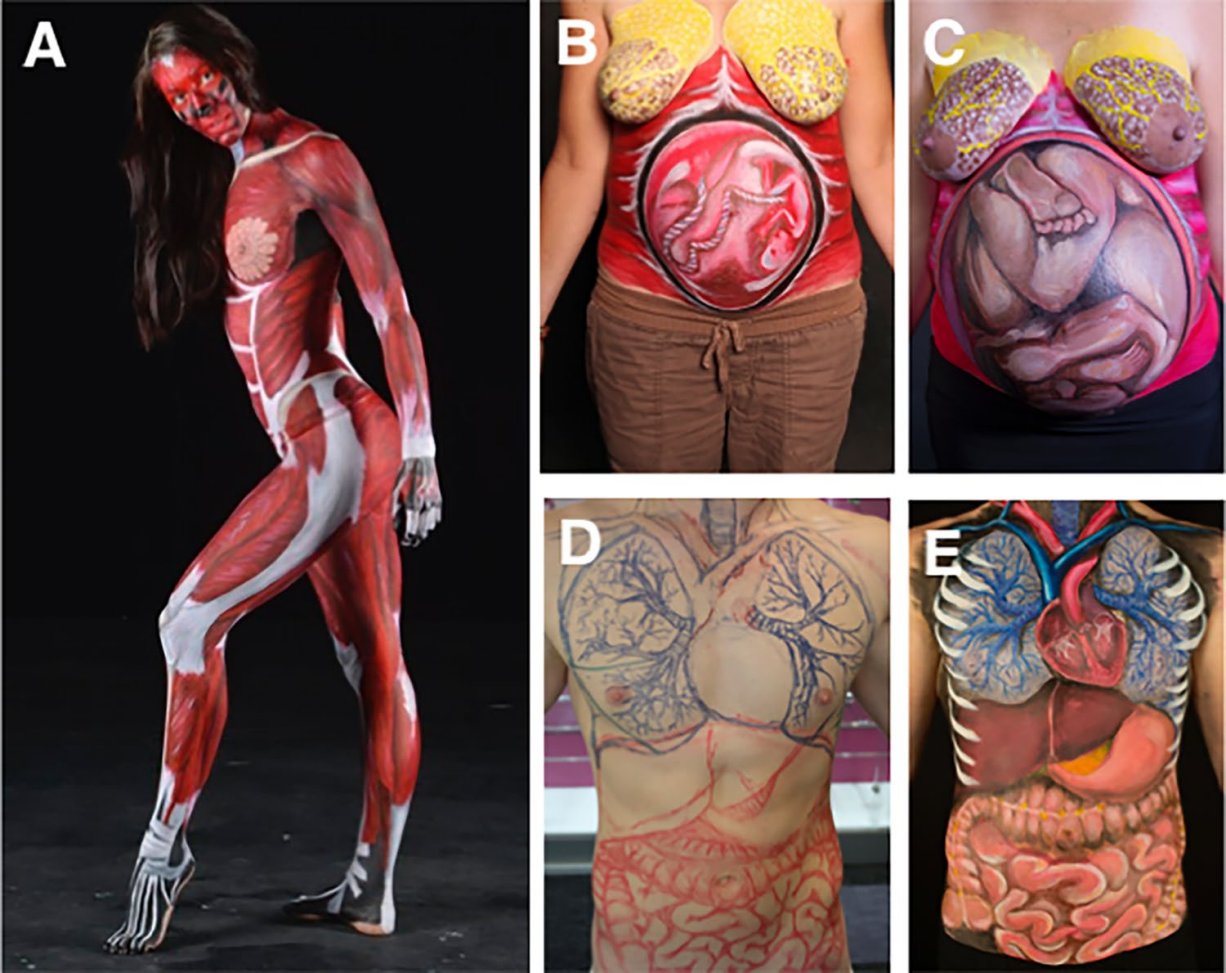
Anatomical Woman displayed muscles of the entire body (**a**) and a Pregnant Woman at 4 months (**b**) and 9 months (**c**) of gestation displayed development of the breasts and abdomen (foetus). Systems Man was completed to display the organs of the thoracic and abdominal cavities through land marking (**d**) and painting (**e**)

**Fig. 5:**
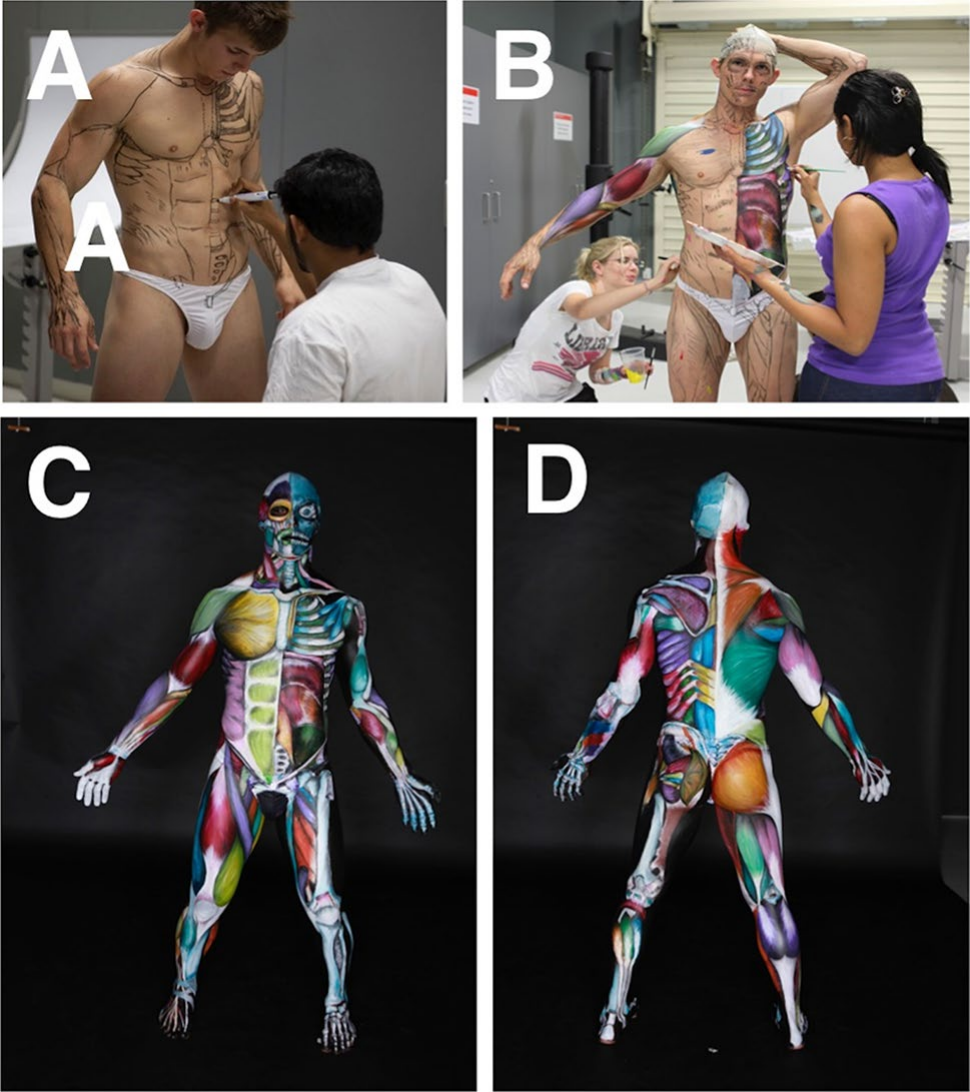
The Multi-coloured Man project took 24 h to complete at JCU. Land marking took 10 h (**a**) and the painting 14 h (**b**). The completed project can be seen: front (**c**) and back (**d**). The superficial muscles were displayed on the right-hand side of the body and the deep muscles on the left-hand side

The “Anatomical Man” projects included the study of both man and woman subjects. These anatomical BP projects were typically run overnight and often took 18 to 24 h to complete. The process of BP was followed precisely to obtain accurate results (Figs. 1, 2, 3, 4, and 5). First, the models were landmarked accurately using pictures and anatomy atlases (Figs. 4 and 5). The land marking was done using black whiteboard markers to accurately delineate all muscles, bones, bony landmarks, organs, blood vessels, and nerves. This phase was important as it created the stencil that was used for the second phase. In some projects, this initial phase of land marking took up to 8 to 10 h to complete. The second phase of each project consisted of painting and shading. Professional face and body paints (Face Paints Australia) were used for all the BP projects as the finished product was long-lasting and did not crack; a range of professional paintbrushes and makeup brushes were also used for these projects. Anatomical pictures from anatomical atlases such as *Atlas of Human Anatomy* by Netter were used as guides for the student artists. At the completion of the painting stage, a student photographer took professional photographs for use as resources, publication, and promotion.

The “Anatomical Man” BP projects complimented the BP carried out by students from week 1 of each course. In anatomy classes, students were encouraged to place importance on the “process” of BP, not on the finished product. In this way, it became a real learning exercise and the artistic abilities of the students were not essential [3, 4, 15]. The process of BP consisted of observation of bony landmarks, form and proportions, palpation of important landmarks, the use of whiteboard markers to outline the important landmarks, drawing in of origins and insertions, the outlining of important organs, and finally the painting. For the “Anatomical Man” BP projects, however, it was important that students had advanced artistic abilities to ensure the precision and accuracy of the finished works.

### Analyses

A SurveyMonkey survey was used to assess the value of these anatomical BP projects for the student participants for several projects. The online survey on student perceptions of the extra-curricular BP projects was offered to participants 1 week after the project. Students were asked ten questions to assess the variables known to predict student engagement regarding BP [39–42], such as enjoyment, use as a learning tool, participation in BP activities, class bonding, assistance in learning anatomy, and overall level of interaction with classmates. Students were also asked to rate (self-assess) how much the BP projects helped them with their long-term, deeper understanding of anatomy. Students rated each variable across a 4-category Likert scale with 1 being the lowest and 4 being the highest-none (1), a little (2), some (3), or substantial (4)-and were asked for comments regarding the activity. Data were coded numerically and entered into SPSS release 19 for Windows. Table 1 describes numerical variables as mean values and standard deviations (https://docs.google.com/document/d/1V48_WY-7NTzdErwz9q-y00duEjHmsKvE/edit?usp=sharing&ouid=109877924874709001981&rtpof=true&sd=true).

**Table 1:** Frequency analyses of students’ views on involvement in body painting projects outside of the classroom (*n* = 31)

BP variables	Mean/4	Standard deviation	Percentage Some/substantial
What was your overall level of enjoyment from participating in the “Anatomy Man” projects?	3.94	0.246	100%
What was the overall quality of your learning experiences from participating in the “Anatomy Man” projects?	3.78	0.491	96.7%
Do you think the “Anatomy Man” projects and body painting will be relevant to the later years of your degree?	3.50	0.88	87.1%
Do you think the “Anatomy Man” projects and body painting will be relevant to your professional career?	3.38	0.942	87.1%
What was your level of accomplishment when completing the “Anatomy Man” projects?	3.63	0.707	93.6%
Do you think participating in the “Anatomy Man” and/or body painting projects has made you feel more confident when later dealing with real patients?	3.34	1.004	80.7%
Do you feel that participating in the “Anatomy Man” projects was a valuable learning experience?	3.81	0.64	93.5%
Do you feel that participating in the “Anatomy Man” and/or body painting projects improved your interactions and bonding with other members of the class?	3.63	0.793	87.1%
Did participating in the “Anatomy Man” and/or body painting projects assist your deeper understanding of human anatomy?	3.66	0.787	93.5%
Did participation in the “Anatomy Man” and/or body painting projects assist your long-term memory of anatomy?	3.59	0.798	93.3%

## Results

The sixteen extra-curricular BP projects carried out were as follows: Anatomical Man (muscles; 4 projects) (Figs. 1 and 2), Skeletal Man (bones; 2 projects) (Fig. 3), Anatomical Woman (muscles; 2 projects), Pregnant Woman (pregnant woman painted once per month until birth) and Systems Man (visceral anatomy, organs; 2 projects) (Fig. 4), Multi-coloured Man (muscles in multiple colours; 3 projects) (Fig. 5), Process Man (4 quadrants to show the process of BP), and Neurovascular Man (half nervous system, half cardiovascular system). Most projects were completed within 24 h; about 8 to 10 h for land marking and 12 to 14 h for painting. An invitation was made to the university community to watch the process, and we had many visits from staff and current students across many programs, not only the ones doing anatomy, also from the Head of School, Dean of Medicine, and Deputy Vice-Chancellor. These visits were important in providing the team with moral support through the long hours of the projects.

Surveys were returned by 31/41 (75.6%) participants comprising medical, dental, osteopathy, biomedical science, and chiropractic students at both institutions. Numerical variables in Table 1 (https://docs.google.com/document/d/1V48_WY-7NTzdErwz9q-y00duEjHmsKvE/edit?usp=sharing&ouid=109877924874709001981&rtpof=true&sd=true) are described as mean values out of 4 and standard deviations, while categorical variables are described as percentages (for the highest categories of some/substantial).

Analyses showed that 93.5–100% of participants enjoyed the projects and considered them a valuable learning experience (Table 1; https://docs.google.com/document/d/1V48_WY-7NTzdErwz9q-y00duEjHmsKvE/edit?usp=sharing&ouid=109877924874709001981&rtpof=true&sd=true). Furthermore, participants rated the relevance of BP to their profession at 80.7–87.1%, level of accomplishment at 93.6%, and interactions with classmates at 87.1%. Student participants reported that participation in the BP projects assisted their deeper understanding of human anatomy (93.5%) and their long-term memory of anatomy (93.3%). Students were very complimentary of the experience and their comments are summarised in Table 2.

**Table 2:** Student comments in SurveyMonkey surveys regarding participation in body painting projects outside of the classroom

“The Anatomy Man projects not only helped to enhance my understanding of anatomy but allowed me to inspire others to view anatomy in a different perspective. It was a great honour being part of such an innovative learning experience and I strongly recommend every student studying anatomy or simple interested in anatomy to give body painting a go!”	“I found that participating in this body painting project helped further my understanding of anatomy, especially to form a 3 dimensional perspective in terms of how each muscle correlates to another. It was very valuable to also do half superficial and half deep muscles as it is often not clear in textbooks which muscles are superficial and which are deep to one another.”
“This project was an innovative way to get students involved in anatomy outside the stereotypical books and presentations. As society changes and we change with society, so too should our teaching and learning methods. Although this type of project may not appeal to every individual, for some it gives them the best opportunity to learn and develop their skills and understanding. I myself struggle to learn from simply reading text, and prefer visual cues to best understand content. Having gotten the chance to be the actual model for skeletal man and get every bone drawn onto myself is something I will never forget! I tell people about this experience all the time and even have a photo of me fully painted in my clinic. It was not only educational; it was rewarding to be part of a project like this that’s taking education to a whole new level. I was self-conscious about my body before this project as well and was nervous at first to take part, but by putting myself out there I have never been happier and more confident. Thanks for a terrific project.”	“The projects brought anatomy out of the text book and into life; visualizing, palpating and painting anatomy onto our models gave me the most insightful prospective that any learning tool has given me thus far. It not only asked of us that we know where each muscle/bone/organ be, but also where they sit relative to one another and how they interact as a whole system. I will greatly value such a unique learning experience along with the wonderful people I learnt from, and collaborated with.” “This is a valuable project and creates an experience of learning more that what a text book could offer. It allows the student to question further their knowledge basis whilst opening a prolonged period of reflection whilst painting the muscles in my opinion. Definitely the ability to prosect and then knowledge basis whilst opening a prolonged period of reflection whilst painting the muscles in my opinion.”
“A great experience, and the skills obtained from both body painting and the Anatomical Man project greatly improved my understanding of anatomy and the real life application of the theoretical knowledge obtained through study.”	“Unbelievable learning activity that also was a lot of fun! Massive thank you to CD and her team for providing the opportunity. A unique approach to learning anatomy has definitely resulted in a better learning experience for me. I truely hope there are more opportunities like this in the future to get involved with, making my learning fun.”
“The ‘Anatomy man’ project was a great project bringing together staff and students from a number of different disciplines, providing an enjoyable yet educational experience. Greatly recommend	“A very enjoyable and worthwhile experience. The greatest feeling was completing a project as a part of a team representative of the unique Anatomical teaching methods at RMIT
It was awesome, can’t wait to do more projects!!!! So much fun and I learnt a lot!!!”	Was a life changing experience that I will never forget!”
“It was a fantastic project to be a part of. Trying to work together with others to create something so amazing and for such a long period of time. I loved doing this and was so proud of how it turned out!”	“It was amazing being involved with the multi-coloured muscle man project and the sense of accomplishment when it was finished made all the hours of drawing and painting worth it! I will definitely be volunteering for future projects too.”
“I felt as though the ‘Anatomy Man’ body painting project was extremely beneficial to myself individually, and that involvement of more participants would be invaluable in terms of the student experience. This experience has enhanced my understanding of the human body as a cohesive biological machine and deepened my interest in human anatomy. From this experience I have obtained a complex and unique view of the human form. I would jump at the opportunity to participate in a project like this in the future.”	“Painting anatomical structures on the surface of the skin helped my understanding in equal measure to using cadavers. It vastly improved my understanding of surface anatomy which is crucial for health practitioners such as chiropractors and osteopaths.”
	“It was a fantastic experience. I feel I gained a lot from participating in the project both on an academic and personal level, and would do it again in a heartbeat!”
“Great opportunity to reflect on my anatomy knowledge.”	“Was a great experience to be part of a major body painting project. Very rewarding and incredible to see anatomy in a different way.”
“Overall it was an incredible learning experience, although very tiring it was really fun to participate in such an amazing project.”	“This was not part of my degree, but I really enjoyed being the first model for this project. The students and teacher involved were very enthusiastic. I learned heaps and had fun doing it.”
“This is a very good way to learn anatomy. It allows you to bring your theoretical knowledge into a practical issue. This helps to visualize and understand the body in a different way and helps you to put in practice the theoretical concept of anatomy in a real body which is useful to understand better a future patient.”	“It was a really good revision tool and it’s main benefit was surface anatomy which is what is being dealt with in any health-care profession dealing with day to day people. It also helps with origins and insertions and visualising the direction of fibres which help learn actions. Lastly it was great fun and good bonding between classmates.”

**Fig. 6:**
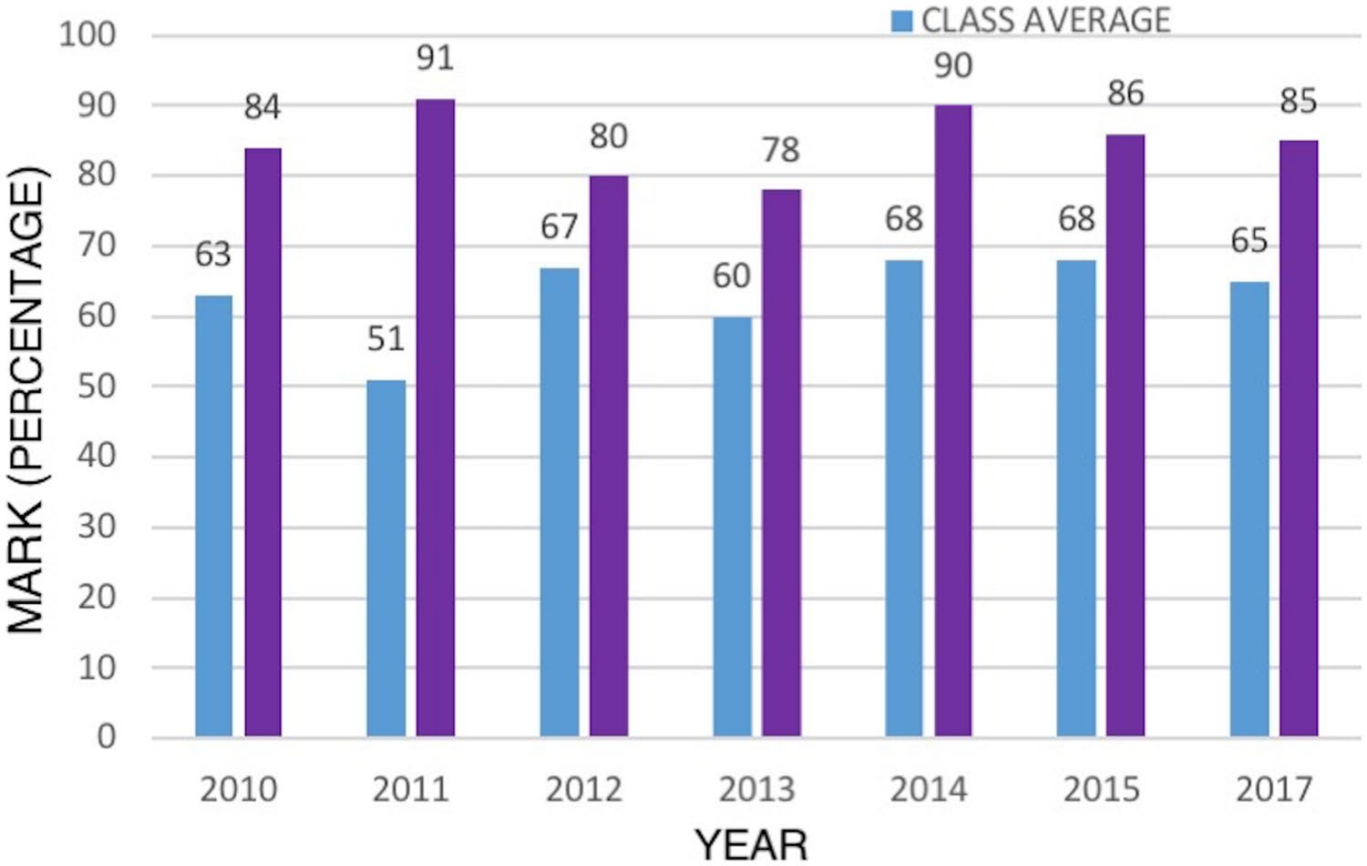
Distribution of average marks for the entire student cohort (blue) and the participants in the BP projects (purple)

The average marks for each year for the entire cohort and the marks obtained by participants of the BP projects at both institutions are shown in Fig. 6 for comparison. Assessment items were similar at both institutions. Class averages ranged from 51 to 68% (average 63%, SD 5.6); while the marks obtained by participants ranged from 67 to 95%, with an average of 84% (SD 4.8).

All finished work was photographed professionally at the end of each project to provide valuable learning resources for teaching and results for publication and promotion. Many projects were also photographed by the press at the finished stage and were reported in major Australian newspapers (*The Townsville Bulletin*, *The Age*, *The Australian*), television and radio, and on websites in over sixty countries all around the world. Furthermore, the work was reported widely on social channels.

## Discussion

This work demonstrated that medical and health science students can be inspired and engaged to learn surface anatomy “outside the classroom” using a self-directed approach. Although many previous BP studies reported regional learning, dedicated to one or two regions per session and under supervision of an academic, the current study is novel as it includes BP the *entire* body, as a living, and dynamic model, with the student team being able to improve their physical examination skills, anatomy knowledge, and engagement as observed by the chief investigator during the projects and self-assessed by participants. These projects presented previously unpublished reports of body painting of the *entire* human body by students with advanced artistic abilities, thus producing learning resources and unintended benefits for the department and the university. The teacher in this study had a reputation for innovative approaches to teach anatomy [3, 4, 15] that encourage students to use innovative approaches to learn anatomy at home (“outside the classroom”), whether BP on relatives or friends or using Play-Doh and whiteboards to learn anatomy. Indeed, in 2020, during online teaching during the COVID-19 lockdown, many students uploaded photographs of their work at home, including BP their children, creating anatomical structures such as the larynx using origami or Play-Doh, or drawing on their cupboards or mirrors [43]. In this study, student participants reported that they found learning outside the classroom, via the extra-curricular BP projects, valuable for improving their palpation and tactile skills, as well as assisting them with deep and life-long learning of human anatomy, consistent with previous reports for BP in class [3, 4, 44]. During these projects, other students (non-participants) were able to observe the participants at work, learn from them, and participating if they desired. The “Anatomical Man” BP projects shocked, inspired, and amazed participating students and other student observers, achieving a very high level of engagement as quantified in Table 1 (https://docs.google.com/document/d/1V48_WY-7NTzdErwz9q-y00duEjHmsKvE/edit?usp=sharing&ouid=109877924874709001981&rtpof=true&sd=true). In the weeks following the projects, we observed an increased number of non-participating students participating in BP in anatomy practical classes, markedly more than in previous years where there were no projects. For example, before the project about five students would BP in a practical class, while after the project, we observed an increase of up to twenty students BP per class. Also, the fact that the projects were carried out by a student team was an effective way of showing these students that everybody is capable of this work, as students were able to see their classmates participating and achieving inspiring results. Students subsequently become very confident and motivated to carry out this learning approach as either models or artists in class. This became evident when we saw several students who were not keen on the BP at the start of the course change their views significantly as the semester progressed and many of these students became active, capable, and enthusiastic participants in BP by the end of the course [15].

Participation in BP projects was popular among students in all the courses. Running extra-curricular anatomical BP projects was inspirational and pivotal in enthusing students and giving them the confidence to participate in BP during anatomy classes and was also a valuable and engaging extra-curricular exercise for those students seeking extra anatomy learning and engagement through the projects. Spending a period of 18–24 h drawing and painting anatomical structures was both educational and inspiring to student participants as it allowed them the opportunity to improve their palpation and tactile skills and their deep learning of anatomy and to establish valuable peer interactions. As can be seen from student comments in Table 2, participants appreciated the interactions with their peers and teacher and they enjoyed working as part of a team in the learning process. Learning outside the classroom in teams allowed students to further develop the self-directed learning skills that were fostered in the classroom by the teacher. Positive peer interactions are known to assist in the development of collaborative learning and motivation for students to learn [41]. Consistent with “engagement theory”, peer learning was an important part of the extra-curricular BP experience for students in this study, as students were engaged in meaningful learning activities involving peer interaction to achieve deep learning [40, 41].

Students who participated in the “Anatomical Man” BP projects reported that this experience was the highlight of their programs: They reported it to be very beneficial for their own learning outcomes in anatomy, particularly in relation to improvement of their palpation and tactile skills, and an engaging and memorable experience. This student-centred approach moved the focus from teaching to learning [45] and encouraged energised and active learning by student participants. The results from this study are consistent with previous reports that students prefer group-based and experiential (“hands-on”) practical learning of anatomy [4, 46, 47]. Experiential learning approaches, like BP, that are stimulating, engaging, and fun have been reported to produce confident and self-directed learners that achieve both deep learning and life-long learning [2–4, 15, 24, 48], while also developing the palpation and tactile skills needed for their professions. Students who participated in the BP projects were the high achievers in the cohort tending to obtain distinctions (75–84%) and high distinctions (85–100%) in the subjects, and although we cannot attribute these final marks unequivocally to participation in the BP projects, we propose that their participation played a significant role in their success. Projects were open for inspection by all students and staff at these universities and in fact we had students from other programs visit during the night-long projects, so we were able to reach a much larger audience than anticipated initially. This work is ongoing and will be offered to health science students at CSU, Albury, Australia, in the coming years.

Running anatomical BP projects outside of the lecture theatre or anatomy laboratory was foundational work that will provide economic development in educational opportunities for learning anatomy and will be a vital approach moving forward for anatomical education. The “Anatomical Man” BP projects carried out by medical, health science, and biomedical science students in their first years at university received considerable media coverage since 2010: newspapers, radio, television, over 441,060 views on YouTube, and university publications. This work was not only used to teach anatomy at university, but was also presented to external groups such as medical specialists such as radiologists from the local hospital and at the British Institute of Embalmers and the Australian Institute of Embalmers conferences in Australia [15].

## Conclusions

Anatomy can successfully be taught outside of the classroom. The extra-curricular BP projects were important foundational work for anatomy education that resulted in a rich experience of self-directed anatomy learning for student participants, encouraging improvement of physical examination and palpation skills, peer learning, high engagement, and assisted deep and long-term learning of anatomy. The self-assessment of improved “long-term memory” by student participants refers to the recall of anatomy over the semester and not into future years or careers. The limitations of this study are that this is self-reported data; students self-assessed the effects of BP on their recall and learning of anatomy. Future work will use longitudinal studies to assess “long-term memory” over longer periods of time (6–12 months), and 12 months plus. These projects using *entire* human bodies were novel and brought anatomy to life, and not only benefited the participants, but also assisted the (non-participating) students studying anatomy courses to observe the process, and to be enthused and inspired to try BP themselves in the practical classes. This study merged human anatomy with art and may inspire other anatomy departments to experiment with student-led extra-curricular body painting to improve student engagement and outcomes. The unintended benefits of this work were that the marketing and media departments of two universities received large amounts of positive feedback.

The extra-curricular BP projects provided an economical means to develop new opportunities for student learning “outside of the classroom”. Considering the increasing significance of online education and the situation encountered by many during COVID-19, this perspective on education will now become more relevant than ever. This work will lead the way with anatomy education post-COVID.
